# Understanding causes of incomplete reports of adverse drug reactions associated with dolutegravir-based HIV treatment in uganda: a qualitative study

**DOI:** 10.1186/s12879-025-11592-0

**Published:** 2025-11-03

**Authors:** Henry Zakumumpa, Helen Byomire Ndagije, Joanitah Atuhaire, Julius Mayengo, Francis Odipiyo, Kiguba Ronald

**Affiliations:** 1 College of Health Sciences, Makerere University, Kampala, Uganda; 2 National Pharmacovigilance Centre, National Drug Authority, Kampala, Uganda

**Keywords:** HIV treatment, Adverse drug reactions, Dolutegravir, Pharmacovigilance, Side effects, Adverse events

## Abstract

**Introduction:**

Although dolutegravir (DTG)-based HIV treatment has demonstrated superior efficacy, it has been associated with adverse drug reactions (ADRs). Robust pharmacovigilance systems that ensure that received ADRs reports are complete and accurate facilitate timely regulatory decision making. However, there is little research seeking to understand the contexts underpinning data capture of DTG- associated ADRs. We sought to understand barriers to achieving completeness of DTG-associated ADR reports in HIV clinics in Uganda.

**Methods:**

We adopted a qualitative exploratory research design. Between August and October 2024, we conducted 36 in-depth interviews with HIV clinicians and eight focus groups with people with HIV or PWH (56 participants) in the HIV clinics of 12 purposively selected health facilities across Uganda. Data were analyzed by thematic approach.

**Results:**

Our study unearthed patient-based barriers to achieving completeness of data in ADR reports which include the six-monthly appointment spacing intervals for PWH which impede recall of events surrounding ADRs, difficulty by PWH in eliciting details on DTG medication (such as brand and batch numbers), non-disclosure of concurrent use of herbal medicines and the fear by PWH of being switched away from DTG-based therapy which was perceived as superior. Health workforce barriers include a novel finding of ‘professional agency’ whereby HIV clinicians manage DTG-associated ADRs at an individual-level, fear of inviting scrutiny post-reporting impedes the supply of complete details on ADR reports such as on dosage as well as the low awareness among mid-cadres such as nurses of the importance of providing complete ADR reports in enabling regulatory action. Health system barriers include the reported shortage of in-puts for conducting laboratory investigations of suspected ADRs and limited incentives to reporters for providing complete information on ADR reports.

**Conclusion:**

Health workers perceived factors underpinning incomplete DTG-associated ADR reports in Uganda as stemming from patient-based factors (e.g. non-disclosure of concomitant use of herbal medicines), health workforce-drivers (e.g. ‘professional agency’ in responding to ADRs) and health system constraints (lack of incentives for reporting). The policy and programming implications for improving data quality in reporting DTG-associated ADRs in Uganda and countries with similar setting are discussed.

**Supplementary Information:**

The online version contains supplementary material available at 10.1186/s12879-025-11592-0.

## Introduction

The management of adverse drug reactions (ADRs) is a global health priority in low and middle-income countries [1].

Robust pharmacovigilance systems are central to reducing the morbidity and mortality of patients associated with ADRs through minimizing risk and maximizing the benefit of medicinal products used in patients [2].

Globally, pharmacovigilance systems are anchored on spontaneous ADR reporting from health workers and patients [3].

The quality of pharmacovigilance data within case summary reports is critical to detecting emerging safety concerns and in supporting remedial regulatory action thereby promoting the safety of medicines [4].

Well-documented ADR reports lend well to multiple ends including facilitating causality assessments at the facility-level, enabling data analysis by National Pharmacovigilance Centres and subsequent regulatory action [4].

Incomplete documentation in ADR reports is a major barrier to rigorous signal detection and analyses for supporting decision making for regulatory action. Incomplete individual case safety reports (ICSRs) are common across the health services delivery landscape in sub-Saharan Africa (SSA) [5]. A recent systematic review by Kiguba et al. (2023) found that in Africa ‘few regulatory decisions on medicines safety are drawn from local data’. Against this backdrop, pragmatic strategies for supporting robust signal detection and analysis are a critical priority given the colliding epidemics of infectious and non-communicable diseases [6–8].

In Uganda, spontaneous or voluntary reporting of ADRs by health workers and patients is still the mainstay of the pharmacovigilance system. The National Pharmacovigilance Centre(NPC) in Uganda tracks ADRs through reports received from frontline health workers through filled paper-based ADR forms available at health facilities countrywide based on the individual initiative of the attending health worker. New digital reporting routes such as the Med Safety App which can be utilized even by patients to report directly to the NPC [9] and social media platforms such as WhatsApp are gaining traction as alternative reporting routes. These reporting options have improved the volume of data received at the National Pharmacovigilance Centre in Uganda. It is Imperative to harness this potential by ensuring better quality reporting to enhance signal detection and subsequently, remedial regulatory action [10–13].

Even when ADR reporting routes in Uganda have been diversified over the past decade, issues around improving data quality to promote signal detection and regulatory action remain largely under-explored.

In this study, we define data quality to refer to the completeness of an individual ADR report whereby all the required information is supplied [4]. We adopted the six mandatory fields for an ADR report to be considered complete [4] ‘among these fields, fields (n = 6 fields) of patient initials and age at onset of reaction, reaction term(s), date of onset of reaction, suspected medication(s): name and details, and reporter information’ [4].

### Dolutegravir (DTG)-based HIV treatment in Uganda

Uganda implemented a national roll-out of dolutegravir-based regimens as the preferred first line treatment option in 2019 [13]. The majority of the 1.4 million people with HIV (PWH) in Uganda have been transitioned from efavirenz-containing to DTG-based regimens [13]. Although DTG has been associated with a range of ADRs such as neuropsychiatric events, gastrointestinal and central nervous system ADRs, novel ADRs continue to emerge hence the need for sustained efforts in strengthening pharmacovigilance systems [14]. Due to the implementation of universal HIV ‘test and treat’ policy, there are PWH newly initiated on DTG-based regimens on a daily basis in Uganda and elsewhere hence the need for constant pharmacovigilance through spontaneous reports of suspected ADRs which are complete and accurate.

With respect to Uganda, there is little research taking an in-depth analysis lens of the factors underpinning incomplete documentation of DTG-associated ADRs [15]. This study was the first of its kind in Uganda to interrogate incomplete documentation of DTG-associated ADRs from the perspective of reporters such as health workers and patients.

The findings of this study will benefit multiple stakeholders including National Pharmacovigilance Centre(s), PWH and their caretakers, health workers, and other relevant regulatory bodies such as technical advisory committees on antiretroviral therapy at the Uganda Ministry of Health.

The study findings potentially have benefits beyond Uganda and are relevant to countries with a high HIV burden given that DTG-based therapy is the recommended first and second-line treatment option globally [16]- [17].

Although there is a steadily emerging evidence base on the facilitators and barriers to reporting of ADRs in Uganda [9, 18] there is little research seeking to understand the experiences of those who report DTG-associated ADRs. While several innovations in reporting ADRs have been developed such as the Med Safety App [9], the quality of data generated has been a barrier to analysis and subsequent regulatory action by the NPC in Uganda. A recently published National Pharmacovigilance Bulletin indicates that the most common missing information in ADR reports in Uganda were, in order of importance; date of reaction onset, date suspect drug was started, the reporter’s email address, concomitant drugs, reporter’s telephone contact, and patient’s age [15].

In Uganda, several alternative reporting routes such as a toll-free telephone line, a WhatsApp platform, and the provision of ADR reporting by email have been successfully rolled out nationally. However, the impediments to achieving good quality data in the received DTG-associated ADR reports at the national pharmacovigilance centre in Uganda, in terms of complete documentation, have not been sufficiently understood. We sought to understand barriers to achieving completeness of DTG-associated ADR reports in HIV clinics in Uganda.

## Methods

### Research design

We adopted a qualitative exploratory research design [14]. We sought to understand facilitators and barriers to achieving completeness of data in reporting DTG-associated ADRs from the perspective of health workers given their operational context, and from the lived experience of PWH with respect to ADRs. In so doing we adopted a ‘constructivist’ paradigm which ‘seeks to understand the complex world through the perspectives of individuals and their unique experiences’ [19].

### Theoretical orientation

This study is broadly informed by the analytical framework proposed by Levesque and colleagues in terms of the multiple dimensions that shape access to health care [20]. The framework incorporates both demand-side factors (such as patient health seeking behavior) and supply-side or health system drivers (such as availability of medicines and commodities) as well as the ‘interconnectedness’ between these dimensions in shaping access to health care. This framework guided us in developing our in-depth interview guides around the multi-level analytical lens of individual-level, health system, community and context factors that influence the reporting of ADRs and the completeness of data remitted to the NPC in Uganda. Further still, this framework informed our data analysis by offering an overarching deductive thematic framework (Table 2). In line with the framework approach to qualitative analysis, we categorized our inductively-generated themes under three broad themes posited as influential on access to health care.

### Study sites

**Fig. 1:**
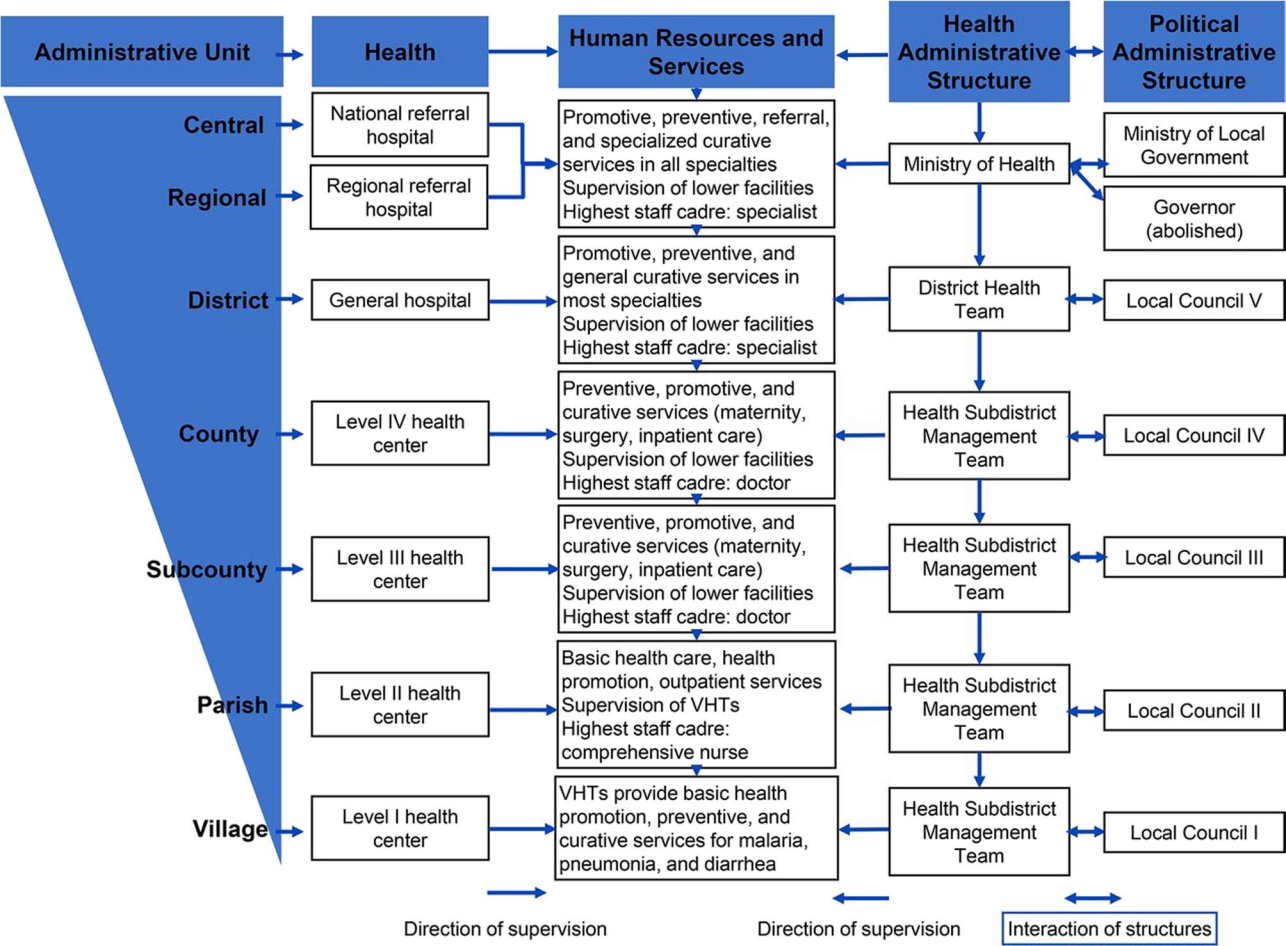
Uganda’s hierarchical health system structure

We utilized purposive sampling to enroll study sites. We sampled sites from each of the four major sub- regions of Uganda. Broadly, we aimed to maximize representation by; a) level of service delivery in the Ugandan health system (tertiary/secondary/primary) (Fig. 1), geographic sub-region of Uganda (e.g. Northern/Western, Eastern and Central) and ownership-type (public/private) [20]. We sought to understand reporter experiences of data capture at the tertiary level of care (regional referral hospitals), secondary level (general Hospitals) and primary level of care (e.g. Health Centre IVs). Figure 1 below shows the different levels of service delivery in Uganda.

In addition, we sought to gain an understanding of health worker experiences by geographic sub-region of Uganda due to varying contexts such as regional variations in the state of physical infrastructure with respect to internet connectivity or the reliability of electricity supply [9]. Hence this study was conducted in four geographic sub-regions of Uganda (e.g. Northern, Western, Eastern and Central).

This study was conducted at twelve facilities across Uganda. At the tertiary level of care (Fig. 1), this study was conducted at four Regional Referral Hospitals (RRHs) representing the four sub-regions of Uganda namely; (i) Lira RRH in Northern Uganda (ii) Mbarara RRH in Western Uganda (iii) Jinja RRH in central Uganda and (iv) Mbale RRH in Eastern Uganda.

At the secondary level of care, this study was conducted at the general hospital in Ntungamo District in Western Uganda, the general hospital in Bududa district in Eastern Uganda, the district hospital in Iganga District in Central Uganda and a private general hospital in Lira District in Northern Uganda.

At the primary care level, we sampled four Level IV health facilities of Ogur HC IV in Northern Uganda, Bwizibwera HC IV in Western Uganda, Maluku HC IV in the Central Uganda and Bugembe HC IV in Central Uganda.

We conducted this study at the HIV clinic in each of the sampled facilities. Over the past five years, antiretroviral therapy has been the leading contributor of ADR reports in Uganda according to the Pharmacovigilance Bulletin of 2022 of the National Drug Authority [18]. The recent national roll-out of DTG-based HIV treatment and anti-tuberculosis medication known as isoniazid preventive therapy (IPT) offered a conducive backdrop for this study [21]. Furthermore, HIV clinics currently provide management of co-morbid conditions such as tuberculosis (TB), hypertension and Type 2 diabetes. Hence, HIV clinics offered us a rich case study of reporter experience in data capture during reporting of ADRs owing to the multiple conditions managed there.

### Study population

In Uganda, health workers are, by far, the leading sources of reports of suspected ADRs received at the National Pharmacovigilance Centre [9]. As such, health workers were the primary participant category we targeted in this study. HIV treatment is the leading source of ADR reports at the National Pharmacovigilance of Uganda [9, 13]. To this end, we sought to understand the perspectives of HIV clinicians who routinely report ADRs based on their occupational experience of attending to PWH in the Ugandan HIV services delivery landscape. In addition, we sampled pharmacists at selected study sites who serve as pharmacovigilance focal persons particularly at the tertiary level of care. There is a growing pivot towards patient-led ADR reporting [18, 22], as such, we enrolled PWH at participating study sites to enact a complete picture of the reporting of ADRs from firsthand accounts by the individuals directly experiencing ADRs.

### Data collection

Data were collected on-site at participating facilities between August and December 2024. We commenced recruitment of human subjects on 30th August 2024. We completed the process of enrolling study participants on 18th December 2024.

Table 1 below shows the category of participants who participated in our study.

**Table 1:** Category of participants

Category of participants	Data collection tool	No. of participants
HIV clinicians	In-depth Interviews (IDIs)	36
Pharmacovigilance focal persons	Key Informant Interviews(KIIs)	06
People with HIV	Focus group discussion (FGDs)	08 (56 participants)

#### Focus group discussions

We conducted four focus group discussions (12 participants in each) to understand the lived experience of PWH with respect to dolutegravir-associated ADRs and its bearing on reporting to the NPC in Uganda either directly by PWH such as via the Med Safety smart phone app which provides a platform for patients to report ADRs or to report via their attending health workers [9]. The focus groups with PWH were led by the first author who has extensive experience in qualitative pharmacovigilance research [23]. The focus groups were conducted in *Rutooro* the local language spoken in mid-Western Uganda. The lead investigator was assisted by six Research Assistants with extensive experience in health services research.

PWH were selected with the help of the HIV clinic in-charge who consulted the paper-based ADRs register at the facility and selected PWH who had reported a suspected DTG-associated ADR over the past twelve months. The selected PWH were invited to participate in the study on a purely voluntary basis. The investigators were guided by the Declaration of Helsinki in handling issues pertaining to HIV status disclosure by PWH, adhering to the highest standards of confidentiality and ensuring anonymity in patient data. On the day of the week when the selected PWH report to the facility for medication refills they were briefed on the study objectives and invited to participate. Those who agreed to participate were requested to provide written informed consent before being enrolled in the study.

#### Inclusion criteria

We included PWH who were 18 years or older. We selected PWH who had a recorded adverse drug reaction in the past twelve months at participating facilities.

#### Exclusion criteria

We excluded PWH who were less than 18 years of age. We also excluded PWH who did not report an ADR over the past twelve months.

#### In-depth interviews (IDIs)

We conducted a total of 24 in-depth Interviews (IDIs) with HIV clinicians to understand their experience in data capture during reporting of ADRs and their preferences of the different reporting routes (such as paper-based ADR forms versus alternatives such as the Med Safety smart phone App).

To secure study participants such as HIV clinicians, we contacted facility in-charges of the 12 purposively selected study sites. A formal letter was addressed to the facility in-charge of each of the selected twelve health facilities. Attached to this letter, was the formal ethical clearance issued by a local institutional review board (The AIDS Support Organization or TASO IRB). After seeking permission to conduct the study at selected sites, we requested them to introduce us to the in-charge of the HIV clinic of the selected facilities. We then sought audience with the HIV clinic in-charge whereby we explained the study objectives and introduced the team of investigators. We then requested the HIV clinic in-charge to nominate the most appropriate HIV clinicians such as those who encounter suspected DTG-associated ADRs in their routine clinical practice. We then invited the nominated HIV clinician to participate in the study on a purely voluntary basis. Face-to-face qualitative interviews with nominated HIV clinicians only commenced after securing individual consent from those willing to participate and explain to them the objectives of the study and invite them to voluntarily participate. In addition, we conducted six Key Informant Interviews (KIIs) with regional pharmacovigilance focal persons drawn from each of the four sub-regions we visited. Pharmacovigilance focal persons provided rich retrospective knowledgeable on trends in ADR reporting and the most common facilitators and barriers in achieving completeness of data on ADR reports. Interviews were conducted in English and were on-site at participating facilities.

### Data analysis

Our qualitative data analysis was informed by the suggested procedures of ensuring rigour in qualitative data analysis as proposed by Miles & Huberman (1994) [24]. More specifically, we utilized the framework approach to qualitative data analysis.

Audio recordings of the interviews and focus groups were transcribed verbatim into text transcripts by a professional transcriber (and later translated into English where necessary).

In the first instance, two investigators (HZ, JA) read the transcripts multiple times for data familiarization. In the second stage, three investigators (HZ, JA, OF) inductively generated codes from multiple readings of the interviews and focus group transcripts. In the third stage, the emergent ‘sub-themes’ were grouped under the dimensions of access proposed by Levesque and colleagues [17] who suggest a multi-level lens entailing; patient-based factors, health workforce factors, health system factors, community and context [17]. This process entailed three investigators (HZ, JA, OF). Hence, we applied a hybrid approach of inductive and deductive theme development [25]. The fourth stage entailed overall interpretation and synthesis [10] involving all investigators (HZ, JA, OF, HN, JM, RK). Disagreements in the assignment of themes and sub-themes were resolved through consensus.

## Results

### Demographic characteristics of health workers

Overall, a total of 42 health workers participated in the study.

More than half of health workers who participated in the study were male (51%) while females were 49%.

The majority of health workers (64%) had a work experience of not less than five years.

In terms of cadre of health worker, the majority were clinical officers (35%) followed by pharmacists (28%), nurses (16%) and medical doctors (9%).

The findings emerging from this study are presented under Lévesque’s’ multi-level analysis of patient-based factors, health workforce factors and health system factors (Table 2) below:

**Table 2:** Emergent themes categorized under levesque’s multi-level analysis lens

Levesque’s dimension of access	Theme	Fleshing out of theme
**Patient-level**	Widespread use of herbs by PWH made it difficult for healthcare workers to distinguish ADRs associated with DTG.	• Older HIV patients over 50 with diabetes or hypertension • Difficulty in conducting causality assessments
	Disengagement due to enrollment in community-based HIV care models	• Appointment spacing of 6 months/multi-month dispensing
	No data on drug/batch number when patients report	• Patients often don’t indicate drug details and other drugs they are taking
	Fear of being switched away from effective DTG-based therapy	• Patients tolerate ADRs to retain effective therapy/trade offs
**Health workforce factors**	Health worker (HW)agency in managing ADRs	• We now know the DTG-associated ADRs no need to report • No feedback/inconsequential to report
	Fear of inviting scrutiny on clinical decision making	• Insecurity in professional competence
	Heavy workloads for HW.	• Long patient queues • Congested clinics • Limited individualized time HW
**Health system**	Shortage of in-puts for laboratory investigations	• Weak lab infrastructure in rural Uganda • Shortage of reagents
	ADR reporting is not incentivized	• Perceptions that reporting is inconsequential • Occupational experience on ADRs reporting is negative
	Little national guidance on ADRs to expect	• Insufficient sensitization on new HIV therapies

### Patient-based factors

#### Widespread use of herbs by PWH made it difficult for healthcare workers to distinguish ADRs associated with DTG

It emerged that there is widespread concomitant use of herbs among PWH at participating facilities. Health workers raised the common use of herbs as a major barrier in establishing an link between suspected ADRs with DTG-based therapy. Health workers indicated that they hesitated to provide complete information such as indicating if there were other drugs PWH were on due to the widespread concomitant use of herbal medicines.

*‘We find it difficult to link the reported side effects with dolutegravir (DTG) because our clients take multiple drugs. Many use herbal medicines concurrently so sometimes we don’t know whether some side effects come out after using the herbal medicines or the ARVs(antiretrovirals). There is a way herbs interact with the HIV drugs they take daily and they end up reacting.”* [IDI, HW, 08].

Health workers reported that there was widespread use of herbs to address the increasing prevalence of hypertension and diabetes particularly among PWH who are 50 years or older. In addition, hyperglycemia has been associated with dolutegravir-based HIV treatment in Ugandan populations [13]. Although the use of herbs for managing hypertension and diabetes was frequently reported across participating facilities, it was particularly pronounced in mid-Western Uganda as a health worker below indicated:

*‘’ There is aggressive marketing of herbal medicines for managing diabetes and hypertension by mobile informal drug sellers. Many patients widely use herbs because they know them to help bring down blood sugar. These people are drawn to trying herbals because they know there is an alternative to modern medication for diabetes which they can’t afford but for HIV*,* they know there is no alternative at the moment.”* [IDI, HW, 02].

*‘’Last year they told me that I have also developed diabetes but I have no money to buy metformin from private retail pharmacies. My grandmother told me that our community has been depending on certain leaves (herbs) to cure diabetes. I have been taking these leaves since last year’* [Male, FGD, 11].

PWH indicated that while antiretrovirals are widely available at public facilities without charge owing to external assistance from PEPFAR and other external donors, when it comes to managing hypertension and diabetes, out- of -pocket payments for medication have become inevitable.

*‘’After I turned 50*,* I was told that besides HIV I now have high blood pressure. Whereas for HIV I am given drugs without charge*,* for managing my high blood pressure*,* I have to dig in my own pocket to pay for hypertension medication because they are frequent stock outs here’’* [Male, FGD, 06].

Although first-line drugs for managing NCDs such as metformin for type two diabetes or hydrochlorothiazide for hypertension are on the World Health Organization’s list of essential medicines, it was reported that they are frequently in short supply at participating facilities. Given the widespread extreme poverty in Uganda, PWH often find they don’t have the money to buy medication for co-morbid NCDs and hence resort to herbal remedies which are deeply entrenched in traditional Ugandan culture.

#### Longer intervals in HIV clinic appointments impeded recall of onset of ADRs

Our focus groups with health workers highlighted barriers to the timely reporting of DTG-associated ADRs by PWH due to the implementation of less-intensive HIV care models in Uganda. Unlike previous HIV treatment delivery which required fixed monthly visits to facilities for review for all PWH, ‘differentiated service delivery’ models in implementation since 2018 in Uganda emphasize reduced contact with the formal health systems for PWH who are stable on ART or those with suppressed viral loads [26]. For instance, multi-month dispensing of antiretrovirals of six months implies that PWH are only required to see a health worker once every six months. Uganda is currently implementing two community-based HIV care models where PWH can access their HIV medication refills from the community or through peers who pick refills from health facilities on behalf of their colleagues [26]. PWH are only required to come for reviews only twice a year or in six-monthly cycles. Health workers indicated that even when PWH experience suspected ADRs, they still report to health workers at their next appointment which could be six months after they have experienced suspected ADRs. It was indicated that patients who belatedly report ADRs are often unable to accurately recall the details such as the onset of symptoms and their duration.

*“Many health facilities are now giving multi-month medication refills of six months. We have seen clients who suffer silently with adverse drug reactions and only report them at their next appointment which is after six months. A client comes to you after six months and they are reporting something which happened six months ago and they struggle to fully recollect all the details you need to report it as an ADR. So*,* how are you going to handle that?”* [IDI, HW, 03].

The delay in reporting suspected ADRs by patients had implications on their ability to recall details such as the onset of the ADRs and their duration. In turn, health workers were unable to report such ADRs to the NPC which requires one to fill over 18 fields for an individual report to be considered complete. This was partly attributed to the phenomenon of incomplete ADR reports where health workers only fill the details they are able to elicit from PWH who often can’t recall all the needed information owing to the passage of time such as six months after the fact.

#### Paucity of details on offending drugs

A common refrain from health workers was that PWH who report suspected ADRs are often unable to provide details on the brand of DTG they were taking or the batch number. These were described as critical details required in an ADR report for it be regarded as complete by the National Pharmacovigilance Centre (NPC) of Uganda without which linking DTG to the suspected ADRs becomes cumbersome.

*“They come and report ADRs to you when it is too late. And now you have to look for a lot of English to write to the NPC. Actually*,* for an adverse event you are required to have reported within 24 hours. They are keeping the problems (of ADRs) at home and by the time they come back for reporting it is already late because they can’t remember when the ADR started*,* the batch number of the drug*,* the other drugs they were taking at the time. I don’t know how we can iron out this problem”* [IDI, HW, 112].

It is important to note that unlike in countries where the majority of patients pay for health care through private health insurance and information on medications is electronically documented, in Uganda the majority of the over 1.4 million Ugandans on HIV treatment are on donor-funded care [10]. External donors in Uganda such as the Global Fund frequently do single source procurements of HIV medications which are then delivered to facilities countrywide [10]. HIV clinicians, let al.one patients, are often oblivious to the details of the drug manufacturer and batch number with the exception of the resident pharmacists in select higher-tier hospitals. Furthermore, in Uganda most of the HIV medicines are generic versions with India being the leading source of the imports.

#### Fear of being switched away from perceived effective DTG-based therapy

Our interviews with health workers revealed that PWH fear being switched away from dolutegravir-based HIV treatment which they perceived as having superior efficacy to existing treatment regimens. Hence, PWH were said to deliberately under report ADRs or provide incomplete information to attending clinicians about the ADRs they are experience with fear that they will be switched to less potent regimens. For instance, we learned that PWH who experience serious DTG-associated ADRs are switched to atanazavar an alternative HIV medication which was perceived as having less therapeutic efficacy by some PWH.

Hence, some patients under-report the severity of an ADR to remain on a regimen they perceive as effective regardless of its adverse events profile and will provide incomplete information to a health worker which impedes reporting. A PWH revealed why she feared to make full disclosure of the true extent of the ADR she was experiencing to her attending clinician.

*‘I have seen some my friends removed from DTG because they complained of many side effects such as diabetes and failing to sleep at night. They were removed from DTG and put on atanazavar. For me*,* DTG is suppressing very well for me but now if I begin telling them that my interest in sex has reduced or that I have numbing in my feet they will put me on a weaker drug’* [Female, FGD, 12].

It was indicated that PWH frequently mask the true extent of DTG-associated ADRs they are experiencing. There was a common perception among PWH that if they disclose the full extent of ADRs they experience then they would compel health workers to switch them away from DTG-based therapy to perceived less effective regimens. It was common for PWH to provide incomplete accounts of ADRs and to tolerate them regardless in order to remain on DTG-based therapy.

### Health worker-related factors

#### Lack of incentives in reporting of ADRs

Lack of feedback emerged as probably the most important disincentive to reporting ADRs by health workers. Complaints of non-receipt of feedback were unanimous across the health workers we talked to. Participants observed that in their professional experience, reporting of ADRs to the NPC is inconsequential in terms of regulatory action from the local National Pharmacovigilance Centre. Several HWs indicated they initially regularly reported ADRs particularly at the start of their clinical careers but were discouraged from continuing to report.

*“Soon after DTG was introduced I received many complaints from clients about lack of libido. I starting sending in reports to NDA (National Drug Authority). We have reported a number of cases to NDA but we have not seen any impact of our reporting. After a while we quit reporting.”* [IDI, HW, 02].

Health workers indicated that even when they bother to report ADRs they are not motivated to fill in all the detail needed to make a complete ADR report because they feel the data is not utilized by the NPC. A section of health workers suggested the introduction of monetary incentives for good quality ADR reports. They argued that incentives have the potential to improve the quality of ADR reports.

*“I think some form of incentives can help improve the quality of ADR reports. We already have monetary incentives under results based financing by ENABLE (Belgian aid program) where we are rewarded directly for providing high quality maternal care. Why can’t similar incentives be provided to health workers with good quality ADR reports?”* [IDI, HW, 01].

The notion of the need to incentivize health workers to attain high quality data in reporting ADRs emerged strongly in our findings. The need for incentives for health workers may be related to underpinning health workforce issues of poor pay.

*“Health workers really need to be motivated. If there is no motivation then it becomes hard for a focal person to organize causality assessment meetings lasting hours. Health workers will expect some monetary incentive after such a long meeting otherwise they will not devote time to it.”* [IDI, HW, 04].

It emerged that knowledge on the importance of pharmacovigilance varies widely by cadre of health worker in participating facilities in Uganda. Whereas pharmacists in particular and clinician cadre (such as physicians and clinical officers) appeared to have some measure of awareness of the importance of reporting adverse drug reactions such as in informing drug recalls, mid-cadres (such as nurses and midwives) did not have the same level of awareness and hence did not appreciate the value of providing complete data on ADR reports as an input for regulatory decision making. The amount of detail required can be a disincentive with lots of fields to fill and congested paper-based forms.

*‘I admit I didn’t realize how important it was to provide all the details required in the paper-based ADR booklets. I will now be more vigilant in reporting going forward’* [IDI, HW, 02].

Fear of inviting scrutiny into their clinical decision making was a common refrain from health workers. Health workers frequently indicated they were afraid of providing accurate data on DTG-associated ADR reports such as on dosage given to patients due to professional insecurities around their clinical judgement calls. In the case of HIV care, very diverse cadres provide clinical care including long-standing recipients of HIV care known as ‘expert patients’ with no prior clinical training. Several of the cadre we interviewed indicated that whenever patients report ADRs they refer these to clinical cadre of the facility in-charges which impacts on the accuracy of data relayed by ‘third parties’.

### Health worker ‘professional agency’ in managing ADRs

A major finding of this study is that health workers in Uganda manage reports received from PWH of suspected DTG-associated ADRs at an individual level and do not bother to relay this information to the NPC or even their colleagues and hence many of the suspected ADRs remain unreported. As such our findings reveal the notion of ‘professional agency’ whereby health workers manage DTG-associated ADRs at an individual-level or make a judgment call on how to handle DTG-associated ADRs without formally documenting the ADR or discussing it with their peers or superiors. For instance, a frequently mentioned notion was that with regard to dolutegravir-based HIV treatment which was introduced in Uganda five years ago, health workers now know from occupational experience that hyperglycemia was one of the common DTG-associated ADRs and they do not bother to report this to the NPC.

Health workers indicated that they assess the severity of a patient complaint of an ADR on their own. They indicated that they frequently take individual decisions such as switching recipients of HIV care away from DTG to alternative regimens such as atanazavar. Hence it emerged that lots of suspected ADRs are unreported and managed at the level of the attending clinician and are not even shared with the wider clinical team at participating facility.

*“They (NPC) do give feedback just that sometimes the feedback comes a little too late*,* because you can report and you wait for two months to receive feedback but by that time you have obviously already managed the ADR yourself. Now*,* what is the use of reporting if the NPC does not guide me on how best to manage the client? I send in a report but you are you are not going to help me manage the client and I have had to manage the client on my own. Why should I bother again to fill a form next time to send to the NPC? We may not see much relevance in reporting so you just decide to ignore and manage on your own.”* [IDI, HW, 04].

Our findings suggest an emerging notion of ‘professional agency’ by health workers in Uganda in responding to ADRs reported by patients where there is no recourse to reporting to the NPC or peer review.

The heavy workload common in congested HIV clinics in Uganda was raised as another impediment to providing all the required data while reporting dolutegravir-associated ADRs. Health workers indicated that they are pressed for time to supply all the required data and that the level of detail is high for an individual ADR report yet they are saddled with long patient queues that are typical at HIV clinics across Uganda. Underpinning health system constraints such as health worker shortages, high attrition rates and frequent staff study leaves mean that staffing strength is often low at HIV clinics and as such the few health workers on the ground prioritize ‘medicines dispensing’ with little time for filling lengthy ADRs forms or even providing a listening ear to complaints from patients.

Some health workers for instance reported that reporting via the Med Safety app is lengthy with cumbersome details required for a single ADR reported. Due to long patient queues health workers reported that they increasingly unable to provide individualized, patient-centered HIV care and as such did not have sufficient time to listen to patient complaints of DTG-associated ADRs to enable them provide the level of detail required while reporting to the NPC in Uganda.

*“You would have a line of 100 clients waiting outside to see you. Then at client number 40*,* one is complaining about reduced libido at which point your mind and body are exhausted and you are not in the mood to entertain long stories. In the morning hours it is different though. We still have a lot of energy to work and we are very receptive and can to listen to your (client) issues but as the day goes on our work becomes more hectic. Picture this*,* you are trying to fill this lengthy ADR form but there is long que grumbling about why you are spending too much time on this single client. So*,* you ignore the (ADR) reporting and go back to clearing the long que. Heavy workloads get to you and change our moods and attitude for listening and being able to record the details we need for an ADR report.”* [IDI, HW, 07].

Although health workers indicated that heavy workloads that are typical in congested HIV clinics influence their ability to provide good quality ADR reports, we asked them which was the preferred route of reporting ADRs if workload was not an issue.

Despite our finding that paper-based ADR forms were the dominant reporting tool at participating facilities, the majority of health workers indicated they preferred to report via the WhatsApp messenger route alternative provided by the NPC in Uganda.

### Health system factors

#### Shortage of in-puts for laboratory investigations

The shortage of commodities for conducting laboratory investigations of reported suspected DTG-associated ADRs was frequently mentioned as an impediment by health workers. Even basic tests were lacking such as blood glucose testing for investigating suspected hyperglycemia in instances where patients complained of excessive frequency of urination after being initiated on DTG. The paucity of consumables needed to conduct laboratory investigations was a common refrain especially in primary care facilities we visited. In Northern Uganda, intermittent electricity supply was a teething problem that rendered laboratories only occasionally operational. Although the regional referral hospitals we visited had comparatively better capacity to process requests from clinicians for examining samples from patients, lower-level facilities were much more constrained. This implied that health workers’ ability to establish a causal relationship between a suspected ADR with DTG which needed laboratory investigations became cumbersome limiting their ability to report or supply all the necessary information to the NPC.

Further still, primary care health facilities reported that they did not have on-site laboratories and that they depend on satellite laboratories which are often located several kilometers away. Uganda runs a laboratory hub system where primary care facilities send samples to a regional laboratory hub system, typically based at a regional tertiary hospital, which often receives samples from multiple lower-level facilities. Dependence on an off-site laboratory has several limitations among which include long turnaround times due to heavy sample loads. A health worker raised the laboratory hub system in Uganda has a barrier to making timely causality assessments of reports of suspected ADRs reported by PWH. It was indicated that without rigourous laboratory investigations, health workers were limited in their ability to report to the NPC and the level of detail they can provide in the standard paper-based ADR forms.

*“For the laboratory services we aren’t fully equipped for some of the tests we use the hub system we normally send and receive results normally after a period of a week or two”* [IDI, HW, 05].

#### Insecurity in professional competence

Several pharmacists participating in our study highlighted insecurities by the workforce in HIV clinics in providing details on ADR reports such as dosage given to patients in cases where they were not sure they had made the right decisions. They indicated that in instances where clinicians are unsure they either didn’t report at all or if they did, they skipped some information on ADR reports (such as the dose given) which they felt that it would invite scrutiny or bring into question their competence as clinicians. For instance, the paper-based ADR form in Uganda requires that the person reporting indicates their name, telephone number and health facility which details were perceived as incriminating for the individual health worker reporting. It is important to appreciate that it is common to find that lay workers provide clinical care in HIV clinics across Uganda due to shortage of physicians in rural parts of Uganda. In some instances, health workers indicated they feared litigation from patients in the event that they had committed medication errors. Indeed, a senior pharmacist at one of the regional referral hospitals opined as follows:

*‘They (clinicians) fear to report because you are inviting examination of your prescription decision making. And indeed in some cases we have re-assessed and found that medication errors were to blame. For instance*,* you are prescribing isoniazid without supplemental pyridoxine so the patient complains of peripheral neuropathy’* [IDI, HW, 08].

Our interviews revealed that medication errors by health workers were not uncommon. It emerged that delay in making causality assessments of some ADRs can have devastating effects on PWH, especially if the patient does not communicate earnestly about the ADR they are experiencing and if the attending clinician is unable to fully grasp the emergent ADR. A health worker recounted a tragic case involving a patient which the attending clinician was unwilling to report to the NPC due to delay in making a link between HIV medication and bone health.

*“I heard of a story of a client who was on TDF (Tenofovir disoproxil fumarate) you know what TDF does is it erodes the bone. This client would come in limping into the facility but because the health worker on was unable to make a correlation that the bone problem was brought on by TDF*,* they kept telling the client to continue swallowing the medicine. The problem was exacerbated. Eventually*,* the client got pathological fractures.”* [IDI, HW, 08]

#### Reporting tools are not user friendly

Health workers frequently mentioned that current paper-based tools for reporting ADRs were not user friendly. Paper-based ADR reporting forms were the most commonly available tool at participating facilities. However, health workers described them as very lengthy, congested with limited space for providing written in-put in some sections, requiring far too much detail than is practical in busy HIV clinics.

Overall, health workers indicated they preferred to report via the WhatsApp route in the future if they were to resume reporting or submit their first report. Health workers indicated they preferred WhatsApp due to multiple reasons. Firstly, they said it was convenient as nearly all health workers we talked to owned a smart phone. Secondly, it provided confirmation that a report had been successfully relayed and the time of dispatch. In addition, it enabled the sharing of photos of patients afflicted by DTG-associated ADRs.

#### Limited guidance on ADRs to be expected

Health workers in our in-depth interviews suggested that recognizing ADRs would be easier if they were sensitized through workshops on the common DTG-associated ADRs to expect. They argued that this would help in recognizing ADRs once patients begin complaining of side effects. The backdrop of the introduction of dolutegravir-based ART in Uganda commencing in 2018 was highlighted as an example of the ‘top down’ decision making that is typical where an ‘upstream ‘policy shift was made to transition away from efavirenz-based HIV treatment to DTG-based ART without much input from frontline clinicians nor trainings around the most common types of ADRs associated with DTG such as hyperglycemia, reduced libido, weight gain and insomnia [13]. A health worker recounted the experience of transition from efavirenz to dolutegravir-based HIV treatment where health workers were not properly guided on the ADRs to watch out for with devastating consequences.

*“They came and just dropped boxes of new drugs (DTG). We were not trained on what to expect in terms of side effects. By the time we knew that DTG was causing elevated blood sugars it was very late. We had lost many people already. People were collapsing deep inside rural villages.”* [IDI, HW, 05].

Participants argued that if the workforce had been brought on board as to what DTG-associated ADRs to expect then the completeness of reporting would have improved.

## Discussion

We set out to understand the factors underpinning incomplete documentation of dolutegravir-associated adverse drug reactions in HIV clinics in Uganda. Our findings reveal multiple barriers which include the six-monthly appointment spacing intervals for stable PWH which impede recall of events surrounding DTG-associated ADRs, difficulty by PWH in eliciting details on the specific brand of DTG implicated in ADRs, the widespread concomitant use of herbal medicines to manage the escalating prevalence of hypertension and diabetes in our sample of PWH and the fear of being switched away from DTG which was perceived by PWH to have superior efficacy. A notable finding of this study is the emerging notion of ‘professional agency’ whereby health workers manage DTG-associated ADRs at their individual-level without providing the complete details on ADR reports if they reported at all. Limited incentives for providing complete information on DTG-associated ADR reports by health workers, fear of inviting scrutiny post-reporting impeded the supply of complete details on dolutegravir-associated ADR reports such as on dosage as well as the low awareness among non-clinician cadre on the importance of providing complete ADR reports in enabling regulatory action which were highlighted as major health workforce-related barriers in data capture. Health system barriers include the reported shortage of in-puts for conducting laboratory investigations of suspected ADRs.

Our study adds to the evidence base implicating health workforce-related influences in the reporting ADRs in LMICs such as the review article by Kiguba and colleagues [6, 15, 16]. Previous studies have highlighted the lack of feedback from national pharmacovigilance authorities as a disincentive to reporting [9]. The finding that resource constraints impedes laboratory investigations in causality assessments at the facility-level has been previously reported [13]. Some studies have explored the subject of providing financial and non-financial incentives to health workers as a strategy for improving the rate of reporting of ADRs [27–29].

### Reporting of dolutegravir-associated ADRs

Although there is an accumulating evidence base from clinical studies regarding dolutegravir-associated ADRs [30, 31], the unique contribution of our study is in providing an in-depth analysis of the barriers encountered in documenting these ADRs from the perspective of frontline HIV clinicians from within their challenging operational contexts as well as from the lived experience of PWH. Our study illuminates the barriers encountered in data capture of DTG-associated ADRs in a country with intersecting crises of a high HIV burden and a weak health system. Our study sheds light on the operational contexts underpinning data capture of DTG-associated ADRs and the factors contributing to incomplete ADR reports.

In this study health workers decried the insufficient guidance provided to frontline health services personnel around the common ADRs to be expected prior to roll out of DTG-associated ADRs. For instance, health workers indicated they were initially oblivious to the link between hyperglycemia and DTG [6]. The findings of this study add to mounting calls for sensitization of health workers of the ADRs to expect prior to the roll out of novel medications particularly those which have been revealed in clinical trials [6]. There are mounting calls in this line around strengthening pharmacovigilance training in the context of roll out of new HIV therapies [32–34] as well as calls for tapping into patient-led ADR reporting [6, 9]. A study in Uganda has piloted a peer support intervention for enhancing the involvement of PWH in reporting ADRs in Uganda [22].

### Programming and policy implications of the study

Although this study reveals that paper-based ADR forms are the dominant route of reporting at participating facilities, the majority of health workers expressed preference for reporting via the WhatsApp messenger route which is a recognized reporting platform by the NPC in Uganda. Sulakhiya and colleagues have explored the use of social media in pharmacovigilance efforts in India [35]. Health workers described current paper-based ADR forms are overly congested and that they do not offer sufficient space for providing complete details on ADRs experienced by PWH which may suggest that further research is needed in this regard in the Ugandan context.

PWH in this study were reported to delay reporting of ADRs to align with six-monthly appointment spacing schedules at HIV clinics [26]. It is important to highlight the danger of late reporting of ADRs particularly in instances where they are life threatening, our study findings point to the need for sensitizing PWH to report ADRs regardless of the appointment schedules issued by HIV clinics. In Uganda, PEPFAR implementing organizations are influential in HIV programming at the sub-national level [26] and they are influential in efforts aimed at ensuring timely reporting of ADRs by PWH in less-intensive models of HIV care such as community-based platforms.

Although the adopted analytical framework proposed by Levesque and colleagues [20], was helpful in unraveling factors underpinning incomplete ADR reports from a multi-level analysis prism of patient-based, health workforce-related and health system factors, our findings appears to suggest some level of interaction between these groups of factors. For instance, we observe that the difficulty by PWH in recalling details on suspected ADRs such as when the onset of symptoms and the batch number of the drugs influenced the level of details that attending clinicians are able to post on paper-based forms. In so doing, our study provides further justification of applying ‘systems thinking’, ‘a mindset that views systems and their sub-components as intimately interrelated and connected to each other’ [30], in pharmacovigilance research which is an under-utilized approach in this content field.

## Limitations

Our study was conducted in twelve HIV clinics in Uganda which could limit the extent of generalizability of our study findings. However, statistical generalizability was not the primary objective of this study. We aimed for an in-depth understanding of the factors underpinning incomplete documentation of dolutegravir-associated ADRs from the perspective of health workers and PWH. Furthermore, we did not interview National Pharmacovigilance Centre officials which would have offered us a more rounded perspective incorporating reporters and the end-users of ADR reports in Uganda.

## Conclusion

Health workers perceived the factors underpinning incomplete DTG-associated ADR reports in Uganda as stemming from patient-based factors (e.g. non-disclosure of concomitant use of herbal medicines), health workforce-drivers (e.g. ‘professional agency’ in responding to ADRs) and health system constraints (lack of incentives for reporting). The policy and programming implications for improving data quality in reporting DTG-associated ADRs in Uganda and countries with similar setting are discussed.

## Supplementary Information

Supplementary material 1.

## Data Availability

The datasets generated during and/or analyzed during the current study are not publicly available due to ethical reasons but are available from the corresponding author on reasonable request.

## Abbreviations

AIDS: Acquired Immune Deficiency Syndrome
ADRs: Adverse Drug Reactions
ART: Anti-retroviral therapy
ARVs: Anti-retrovirals
DTG: Dolutegravir
MOH: Ministry of Health
PEPFAR: The Presidents' Emergency Plan for AIDS Relief
PLHIV: People Living with HIV
SSA: Sub-Saharan Africa
TLD: Tenofovir, Lamivudine, and Dolutegravir
TLE: Tenofovir, Lamivudine, and Efavirenz
WHO: World Health Organization
