# Supplementary material for: Understanding causes of incomplete reports of adverse drug reactions associated with dolutegravir-based HIV treatment in uganda: a qualitative study
Source: BMC Infect Dis. 2025 Nov 3;25:1475. doi: 10.1186/s12879-025-11592-0 (PMC12581420; doi:10.1186/s12879-025-11592-0)
Supplement: Supplementary file 1 — Supplementary material 1. [file 12879_2025_11592_MOESM1_ESM.docx]

**APPENDIX A’**

**IN-DEPTH INTERVIEW GUIDE WITH HEALTH WORKERS**

1. To begin with, please tell me a little about yourself?
2. What do you do in your current role?
3. How long have you been involved in providing HIV care and treatment?
4. What is the nature of HIV services offered at this facility?
5. How many active patients on ART do you currently have at this facility?
6. Please tell us about what you know about pharmacovigilance or the reporting of side effects of drugs?
7. How do health workers approach complaints of side effects by patients at this facility?
8. What is your personal experience in reporting side effects of HIV medicines?
9. What routes of reporting side effects are used at this facility (***Probe:*** *paper-based forms, WhatsApp, email, toll free line, med safety app*)
10. Which is your preferred route of reporting side effects?
11. Why do you prefer to use that route for reporting side effects?
12. Which challenges do health workers experience in reporting side effects of medicines? (***Probe:*** *workload, time pressures, difficulty recognizing ADRs, lack of feedback)*
13. How can health workers be supported to provide complete and accurate reports on side effects of medicines to the National Drug Authority?
14. What is your opinion on the ability of health workers to determine the accurate cause of a side effect of a drug? (***Probe:*** polypharmacy, inadequate competence in pv, concurrent use with herbal drugs, time to onset)
15. What is needed to enable health workers determine that the side effects reported by patients are actually caused by a specific drug they are taking?

**APPENDIX B’**

**FOCUS GROUP DISCUSSIONS GUIDE WITH PATIENTS**

**Form1: Patient Participant Demographic Information Survey**

Today’s Date: _________________Facility Name: ____________________________

What is your age?

1. 18-24 2. 25-34 3. 35-44 4. 45-54 5. 55-64

What is your sex?

1. Male 2. Female

Marital Status?

1. Single 2. Married 3. Divorced 4. Widowed

Differentiated Service Delivery Model

1. CCLAD 2. FTDR 3. FBIM 4. FBG 5. CDDP

How long have you been taking ART?

1. 1-2 years 2. 3-5 years 3. 6-8 years 4. 9-11 years 5. More than 11 years

How do you travel to the ART clinic?

1. Walk 2. Bicycle 3. *Matatu* /taxi 4. Drive

Which ARVs combination are you currently taking?

1. TLE 2. TLD 3………………………

How frequently do you visit the ART clinic?

1. Monthly 3. Every 3 months 3. Once in 6 months 4.

When did you start taking DTG (Dolutegravir)

1. 2018 2. 2019 3.2020 3. 2021 4. Not yet on DTG.

Did your ART Clinic conduct a blood sugar test before enrolling you on DT

G?

1. Yes 2. No.

**Form 2: Patient Focus Group Guide**:

**Instructions:**

•The interview should be held in a private, quiet location.

•The interview should be conducted by a team of two: one who will lead the interview, and one who will take notes during the interview.

•A device needs to be available to record the interview, with extra batteries available in case they are needed.

•Before starting the interview, the client needs to have signed the Written Informed Consent Form:

Verification of informed consent: 1. YES 2. NO

**Introduction:**  Hello my name is …………. Thank you for agreeing to talking to me. I am here as part of the research study team from Makerere University, College of Health Sciences and National Drug Authority (NDA) conducting a study to better understand patient experiences in reporting of side effects of drugsand what factors help or hinder them from reporting these side effects to the National Drug Authority. As a patient who is taking HIV medication your thoughts, experiences and preferences on how you can report side effects are important in helping the National Drug Authority and Ministry of Health monitor the safety of patients in especially during introduction of new HIV medicines and in providing Drug Safety advisories to Health facilities. Your suggestions are important in enabling the NDA help other patients like you taking these medicines.

1. To begin with, please tell me a little about yourself?
2. How long you have been on ART?
3. What do you do for a living?
4. Please tell me about your personal experience being started on DTG.
5. How did your body respond to DTG and IPT?
6. What things did you like about DTG after you were started on it?
7. What things did not go well for you after switching to DTG, if at all?
8. Which side effects did you experience with DTG and IPT, if at all?
9. How long did these side effects last, if at all?
10. What did you do when you experienced these side effects?

***Probes:*** Did you inform the peer leader, informed the health worker, were you switched back to TLE, self-referred myself to another health facility.

1. Which routes of reporting side effects are you aware of ?( ***Probes*** *:*e.g. paper-based forms, med safety app, whatsapp etc.)
2. Have you ever reported a side effect of a drug you were taking? If so, how did you go about it?

What are the barriers to reporting side effects of HIV medicines by patients at your facility? ***Probes:*** shortage of materials, difficulty recognizing side effects, stigma

1. What are your personal preferences for reporting these side effects? (***Probes:*** Peer, HW, paper-based form, med safety app)
2. Which obstacles do patients encounter in filling out the required details while reporting side effects of ARVs?
3. What suggestions do you have to the National Drug Authority and Ministry of Health regarding how patients can be helped to report side effects to the National Drug Authority?
